# Examining Distinctive Working Memory Profiles in Chinese Children With Predominantly Inattentive Subtype of Attention-Deficit/Hyperactivity Disorder and/or Reading Difficulties

**DOI:** 10.3389/fpsyg.2021.718112

**Published:** 2021-10-25

**Authors:** Kean Poon, Mimi S. H. Ho, Li-Chih Wang

**Affiliations:** ^1^Department of Special Education and Counselling, The Education University of Hong Kong, Tai Po, Hong Kong SAR, China; ^2^Department of Special Education, National Tsing Hua University, Hsinchu, Taiwan

**Keywords:** attention-deficit disorder, children, dyslexia, inattentive subtype, reading difficulties, working memory

## Abstract

Although evidence has shown that both RD and ADHD-I children suffer from working memory problems, inconsistencies in impaired modalities have been reported. This study aimed to (1) compare the three WM domains (i.e., verbal WM, visual-spatial WM, and behavioral WM) among pure ADHD-I, pure RD, comorbid ADHD-I+RD, and typical control groups and (2) examine the impact of comorbidity on the three WM domains. A Chinese sample of participants from Hong Kong included 29 children in the ADHD-I group, 78 children in the RD group, 31 children in the comorbid group (ADHD-I+RD), and 64 children in the TD control group. All participants completed the assessments individually. The findings showed that the children with ADHD-I and/or RD exhibited diverse cognitive profiles. In particular, RD was associated with verbal and visual-spatial working memory deficits, while ADHD-I was associated with behavioral working memory deficits. Interestingly, the comorbid condition demonstrated additive deficits of the two disorders but with greater deficits in behavioral working memory. These findings support the cognitive subtype hypothesis and provide a clearer picture of the distinctive working memory profiles of different groups, allowing for the development of intervention programs in the future.

## Introduction

### Attention-Deficit/Hyperactivity Disorder and Reading Difficulties

Attention-deficit/hyperactivity disorder (ADHD) is one of the most common neurodevelopmental disorders in children worldwide, with 8.4% of school-aged children having ADHD [American Psychiatric Association (APA), 2021]. According to the Diagnostic and Statistical Manual of Mental Disorders (DSM-V), ADHD can be divided into three subtypes: the predominantly inattentive subtype (ADHD-I), predominantly hyperactive/impulsive subtype (ADHD-H), and combined subtype [ADHD-C; American Psychiatric Association (APA), 2013]. A recent meta-analysis revealed that ADHD-I is the most common ADHD subtype, followed by ADHD-H and ADHD-C (Ayano et al., 2020). Characterized by a range of behavioral problems, such as difficulty attending to instructions, focusing on tasks, and keeping up with tasks following instructions, the ADHD-I subtype differs from the more commonly recognized ADHD-C subtype, in that symptoms of hyperactivity and impulsivity are minimal or absent (Hurtig et al., 2007). Some evidence suggests that children with ADHD-I may be more academically impaired than those with the ADHD-C subtype (e.g., Weiss et al., 2003; Trampush et al., 2009).

In fact, children who exhibit purely inattentive behavior are likely to underachieve in reading (Warner-Rogers et al., 2000; Willcutt and Pennington, 2000) and have a significantly higher rate of comorbid reading difficulties (RD) than in any other developmental disorder (Turker et al., 2019; Karr et al., 2021). These RD occur despite normal intelligence and a lack of sensory impairment, brain damage, or environmental deprivation (McBride-Chang, 1995; Catts and Kamhi, 2005) and have a global prevalence rate of 7% in children (Dyslexia International, 2021). The comorbidity of ADHD-I and RD in children is 31–45% (DuPaul et al., 2013; Children and Adults with Attention-Deficit/Hyperactivity Disorder (CADD), 2021), an estimate that exceeds the expected chance occurrence (Mueller and Tomblin, 2012). Furthermore, this overlap occurs in both community and clinical samples, suggesting that it is not a selection artifact (Willcutt et al., 2001). More importantly, individuals with comorbid ADHD-I and RD are a group that is understudied. Given the high prevalence rate and developmental challenges this group faces (Bloom et al., 2005), the current study aimed to explore the core neurological deficits of ADHD-I and RD and understand the impact of comorbidity on these shared deficits.

### Working Memory Deficits: Construct and Measurements

Extensive evidence has shown that both RD and ADHD-I children suffer from working memory (WM) problems (Schweitzer et al., 2006; Fostick and Revah, 2018). WM is a multicomponent system providing temporary storage of information for brief periods of time that can be used to support ongoing cognitive activities (Baddeley and Hitch, 1974; Baddeley, 1983, 2012; McCabe et al., 2010). A large body of research has focused on WM deficits in individuals with difficulties in reading (e.g., Gray et al., 2019; Kofler et al., 2019) and attention (Barkley, 1997; Martinussen and Tannock, 2006). For instance, Engle (2002) suggested that WM is highly related to reading, as it helps to maintain or suppress information related to word processing, such as segmentation and blending. At the same time, WM is the capacity for controlled and sustained attention in the face of interference or distraction, which is the ability children with attention-deficit lack (Shipstead et al., 2014). Research has also identified that WM, rather than inhibitory control, is the primary cognitive deficit in ADHD-I vs. ADHD-C (e.g., Diamond, 2005; Huang-Pollock and Karalunas, 2010).

The limited capacity of WM varies widely among individuals. The cognitive and behavioral profiles are the two major directions for understanding individual differences in WM. The cognitive profile is mostly based on the multicomponent model (Baddeley, 2003), which conceptualizes WM as a system with three major components: a central executive component, a phonological loop, and a visuospatial sketchpad (Cocchini et al., 2002; Baddeley, 2012). Both the phonological and visual components belong to the executive component and are referred to as “slave” systems because they hold information for very short periods of time. Considering the entirely different input sensory of the two components, individual differences in WM capacity are assessed by separate techniques that are designed to impose significant concurrent demands on both processing and storage (e.g., Daneman and Carpenter, 1980). In tests of phonological or verbal WM, the participant is required to recall sequences of verbal material, such as digits, words, or non-words (Green et al., 2012). Visuospatial WM tests, on the other hand, involve the presentation and recall of materials, such as sequences of tapped blocks or filled cells in a visual matrix (Alloway et al., 2008b).

Other studies have adopted behavioral measures, such as the Working Memory Rating Scale (WMRS; Alloway et al., 2008a) and the WM subscale of the Behavior Rating Inventory of Executive Function (BRIEF; Alloway et al., 2009; Beck et al., 2010) as behavioral profile measures. Some research (e.g., Shakehnia et al., 2021) has used behavioral measures to evaluate children’s WM within their lived environments (i.e., at home or at school) with information gained from parents or teachers. For instance, questions were asked about forgetting lengthy instructions, missing letters or words in sentences, and frequently making careless errors. Interestingly, some studies have shown that performance measured by a behavioral scale did not indicate concordance with the direct cognitive measures of the two slave systems (Sullivan and Riccio, 2007; Biederman et al., 2008), suggesting it might not evaluate the same skills as those measured by direct cognitive tests. Other studies have suggested that behavioral rating measures and conventional direct measures of WM are significantly related (e.g., Alloway et al., 2008b). Undoubtedly, the combined use of the cognitive and behavioral profiles would provide valuable complementary information to improve our understanding of WM in children with these two disorders.

### WM Deficits in ADHD-I and/or RD Using Cognitive vs. Behavioral Measurements

Although evidence has shown that both RD and ADHD-I children suffer from WM problems, inconsistencies in the impaired modalities have been reported. Previous epidemiological studies have suggested that ADHD-I is likely to be associated with poorer verbal (Wu et al., 2006; Ferrin and Vance, 2014) and visual-spatial WM (Brocki et al., 2007; Cockcroft, 2011; Dovis et al., 2015). However, others have failed to replicate the results (e.g., Geurts et al., 2004; Jonsdottir et al., 2005; Kibby and Cohen, 2008). Nevertheless, the studies that used behavioral measures were more consistent in their findings that children with ADHD-I exhibited a range of behavioral WM deficits (Alloway et al., 2010; Cockcroft, 2011; Holmes et al., 2014; Dovis et al., 2015), especially concerning distractibility and the inability to focus their attention in the face of interference (Engelhardt et al., 2010).

Verbal WM deficits, on the other hand, have been associated with children with RD (Gathercole and Pickering, 2000; Baddeley, 2003; Gathercole et al., 2003; Alloway et al., 2009). For instance, poor verbal WM has been shown in logographic language (Ho et al., 2004; Chan, 2018; Chen et al., 2018) and alphabetic languages (Lehtola and Lehto, 2000; Swanson and Jerman, 2007; Pham and Hasson, 2014). Previous research has yielded mixed results for visual-spatial WM in children with RD (Pham and Hasson, 2014). Regarding behavioral WM, studies using the BRIEF scale have shown that children with RD tend to have difficulties associated with reading-related WM, such as short attention spans, struggling with tasks that have more than one step, and recalling only the first or last when given three things to do (Daucourt et al., 2018; Akyurek and Bumin, 2019). In general, studies have shown individuals with RD to exhibit significantly weaker verbal WM than visual-spatial WM (Bayliss et al., 2005) and behavioral WM (Savage et al., 2007), suggesting that verbal WM plays a more significant role in reading.

When examining the impact of comorbidity, many studies have confirmed that cognitive deficits seem to be more severe in the comorbid group (de Jong et al., 2006). Martinussen and Tannock (2006) revealed that children with ADHD-I and RD were impaired in both verbal and visual-spatial domains of WM. A study conducted by Katz et al. (2011) showed that children with both disorders had more difficulties on virtually all cognitive measures of WM than individuals who had pure disorders. To date, no study has compared the cognitive and behavioral profile of WM among the three subgroups or explored the impact of comorbidity ADHD-I+RD on the comprehensive profile of WM.

To understand the impact of comorbidity, three mutually exclusive hypotheses reign. Early studies proposed the “phenocopy hypothesis,” according to which one disorder might produce symptoms of the other. For example, Pennington et al. (1993) concluded that RD and ADHD may be a phenotype of RD, in that RD causes symptoms in patients with RD and ADHD, rather than ADHD. The second hypothesis is the “common etiology hypothesis,” according to which distinct cognitive deficits appear in groups with pure disorders, while the comorbid group manifests the additive set of symptoms. This hypothesis suggests that the ADHD+RD group shares the basic characteristic impairments of executive dysfunction with the ADHD-only group and impairments in reading-related cognitive functions with the RD-only group. Some evidence has supported the common etiology hypothesis and suggested that the comorbid ADHD+RD group shares the common cognitive risk factors and genetic underpinning of the pure groups (e.g., Willcutt et al., 2005; Gooch et al., 2012). Finally, the “cognitive subtype hypothesis” proposes that the interaction between two pure disorders results in a unique form of cognitive impairment in the comorbid group. In other words, neuropsychological deficits in the ADHD+RD group are different from the simple additive combination of deficits associated with children with either RD or ADHD only. The most recent findings support this hypothesis. For example, Poon and Ho (2014) reported that the ADHD+RD group displayed a greater problem of executive control than the RD-only or ADHD-only groups. Moreover, Wang and Chung (2018) reported that Chinese children with comorbid ADHD+RD displayed greater deficits in auditory WM than did the pure groups. In sum, these three controversial hypotheses are still being tested, and the nature of children with comorbid ADHD and RD symptoms remains unclear (de Groot, 2015). A possible explanation for this inconsistency may be the various abilities and skills involved in different studies that produce complicated results. Interestingly, almost all of these studies focused on the ADHD combined subgroup, leaving the most commonly seen ADHD-I subtype underexplored. The present study addressed this research gap by investigating the shared cognitive deficit, WM, in the ADHD-I, RD, and ADHD-I+RD subgroups and exploring the impact of comorbidity.

### Aims of the Study

The purposes of this study were as: (1) to compare the three WM domains (i.e., verbal WM, visual-spatial WM, and behavioral WM) among the pure ADHD-I, pure RD, comorbid ADHD-I+RD, and typical control groups and (2) to examine the impact of comorbidity on the cognitive and behavioral WM domains. Based on previous research, it was hypothesized that children with ADHD-I would be associated with deficits in behavioral WM, while children with RD would be associated with deficits in verbal WM. It was also hypothesized that the comorbid group of ADHD-I and RD would exhibit severe forms of WM deficit shared by the pure groups, which supported the cognitive subtype hypothesis.

## Materials and Methods

### Participants

Two hundred and two primary school students (*M*_age_=9.3; *SD*=1.1) were recruited from four primary schools in Hong Kong. This sample size was estimated using G*Power (Faul et al., 2009), with Cohen’s *f* (Cohen, 1988) medium effect size of 0.25, an alpha level of 0.05, and 80% statistical power. In Hong Kong, there are approximately 7.4 million people and 8.2% of whom are children aged between 6 and 11years (Census and Statistics Department, 2021). The prevalence rates of ADHD-I and RD are 6.4% (Liu et al., 2018; Hong Kong Association for AD/HD, 2021) and 9.7%, respectively (Child Assessment Service, Department of Health, 2017). A multi-stage sampling procedure was used to obtain the sample. Mass invitations were sent to all public primary schools in Hong Kong and schools to be studied were then randomly selected such that the proportion of students in each selected district represented the number of students in that area. This procedure resulted in the selection of four schools, respectively, with one from Hong Kong Island, another from Kowloon Peninsula, and two from the New Territories. As schools were given the option to withdraw from participation, a further four schools were selected by the same procedure as a backup. Schools that withdrew from participation would be replaced by schools on the back-up list from the same district. Of the schools originally selected, none withdrew from participation. Only children who had been formally diagnosed with ADHD and/or RD prior to the beginning of this study were recruited to participate in this study. The inclusion criteria were as: (1) aged between 6 and 11; (2) overall IQ score above 80; (3) a clinical diagnosis of ADHD and/or RD from a clinical or educational psychologist or a psychiatrist; and (4) native speaker of Cantonese. The exclusion criteria were as: (1) suspected brain damage, neurological, sensory, or other psychiatric disorders. After screening, the final sample included 29 participants (*M*_age_=9.6; *SD*=1.1; age range: 7–11years) in the ADHD-I group, 78 participants (*M*_age_=9.1; *SD*=1.0; age range: 7–11years) in the RD group, 31 participants (*M*_age_=9.7; *SD*=1.3; age range: 7–11years) in the comorbid group, and 64 participants (*M*_age_=9.0; *SD*=1.0; age range: 7–11years) in the TD group. All of them had normal intelligence (≥80) and no suspected brain damage or neurological, sensory, or psychiatric problems.

### Procedures

Ethics approval was obtained through the first and second authors’ institution prior to the commencement of data collection. Before collecting data, the authors contacted the school principals to request their permission to approach the primary school students who are interested taking part in this research. All participants were informed of the research objectives, procedures, and confidentiality of the information obtained from the participants and anonymity. Informed consent forms were then given to the parents and children to obtain their consent to participate. Upon receiving the signed consent forms from the parents, their children completed the cognitive assessments during the screening and assessment phases.

### Screening Phase

Students with ADHD-I and RD diagnoses were identified based on the comprehensive psychological reports conducted by clinical or educational psychologists or psychiatrists. The comprehensive reports include results from the standardized psychological assessments on students’ abilities in reading and writing {e.g., The Hong Kong Test of Specific Learning Difficulties in Reading and Writing for Primary Students—Third Edition [HKT-P(III)]} (Ho et al., 2016) as well as their specific ADHD symptoms (e.g., performance on the Conners Continuous Performance Test; Conners et al., 2018). The authors obtained the relevant information from the schoolteachers after parents gave consent. All students had a formal clinical diagnosis of ADHD-I and/or RD. Those who were diagnosed with ADHD-I were on medication. The paper-and-pencil version of Raven’s Standard Progressive Matrices (Raven et al., 2000) was then administered to the participants to rule out any intellectual disability. The entire screening phase took approximately 30min.

### Assessment Phase

During the assessment phase, the third edition of the backward digit span subtest from the Wechsler Intelligence Scale for Children (WISC-III; Wechsler, 1991) and the visual-spatial WM test from the Cambridge Neuropsychological Test Automated Battery (Cambridge Cognition Limited, 2021a) was administered to the students individually. Their parents were also asked to fill out the WM subscale of the BRIEF (Gioia et al., 2000). The individual assessments lasted approximately 30min. A detailed description of all cognitive tasks is provided below.

### Measurements

Participants’ demographic background information (e.g., age, sex, and education level) was collected using self-report. A comprehensive assessment battery of WM tasks was also administered to the participants, and data on reading performance and behavioral outcomes were collected from multiple informants (i.e., parents) using questionnaires.

#### Intellectual Ability

Raven’s Standard Progressive Matrices (Raven et al., 2000) were used to assess general intellectual ability. This test contains 60 items in total, and each item consists of a visual pattern with a missing piece; participants were asked to identify the correct piece to fill in the missing part and complete the pattern; and those who scored below 80 were excluded from the present study. The Raven’s test has good internal consistency, with a Cronbach alpha coefficient reported of 0.88 (NCS Pearson, Inc, 2007).

#### Behavioral WM

The BRIEF is a standardized measure that allows the observers (i.e., parents) to rate the behavioral measure of WM in children with ADHD. It is a well-researched instrument that provides researchers with a comprehensive assessment tool that results in reliable and valid data (Hendrickson and McCrimmon, 2019). The WM subscale of the BRIEF in Chinese (Gioia et al., 2000) comprises a parent questionnaire designed to assess executive functioning in the home environment. The WM subscale consists of 11 items. Parents were asked to rate their child’s behavior on a three-point Likert scale ranging from 1 (*never*) to 3 (*often*). The internal consistency was 0.80–0.98, and the test-retest reliability was 0.82, indicating good reliability of the BRIEF (Gioia et al., 2000).

#### Verbal WM

The backward digit span subtest from the WISC-III (Wechsler, 1991) was employed to measure verbal WM capacity. This is one of the most common tools used by researchers (Cheung et al., 2014; Siquara et al., 2018) for the evaluation of memory capacity in children aged between 6 and 16years, and age-specific norms are provided. The participants were asked to repeat the orally presented digits in reverse order from two to nine. They must maintain auditory information and select relevant information from irrelevant information to recall orally presented digits in reverse order. Age-standardized scaled scoring was used as the outcome measure. The subset of backward digit span has obtained good reliability, with alpha coefficient of *α*=0.80 (Chen and Hung, 2004).

#### Visual-Spatial WM

The spatial WM test was selected from the Cambridge Neurophysiological Test Automated Battery (CANTAB) to measure visual-spatial WM. It has established a large normative data set and has been widely used in research studies, with over 2,400 peer-reviewed papers supporting its use (Cambridge Cognition Limited, 2021b). The test is a computerized standard measure that begins with several boxes on the screen. The participants were asked to search for a yellow token hidden in one of the boxes. In each search, only one token can be found. When the token is collected, participants must search for another until all have been found (the number of tokens is equal to the number of boxes). The token never appears in boxes where one has previously been found. The number of boxes increases from a minimum of four to a maximum of eight when the difficulty level increases. The variable “between error” counts the number of times participants mistakenly search for a box where a token has been found before. A higher number of between errors represents a weaker ability in visual-spatial WM. This CANTAB test falls within an accepted level of test-retest reliability of 0.75–0.80 (Lowe and Rabbit, 1998).

### Data Analysis

Several statistical methods were employed using the Statistical Package for Social Sciences (Version 26.0; IBM Corp, 2019). The demographic characteristics of the participants were investigated using chi-square tests for independence (for nominal variables) and a one-way ANOVA (for continuous variables), followed by post-hoc tests for group comparisons. To achieve the overarching aim of the study, a 2 (ADHD-I vs. non-ADHD-I)×2 (RD vs. non-RD) multivariate analysis of covariance (MANCOVA) was used, with the three outcome measures serving as dependent variables, and age and IQ serving as controlling variables. A MANCOVA was used to maximize the power to detect significant effects, Pillai’s trace was used to determine statistical significance when comparing three levels, and sample sizes were unequal across cells. When justified by significant interaction effects in the MANCOVA, simple effect analyses were followed by group comparisons using Tukey’s test to control the probability of committing a Type 1 error.

## Results

With reference to the recommendations by Tabachnick and Fidell (2019), the data were scanned for univariate and multivariate outliers to ensure the accuracy of the data file. No outliers were detected. Standard skewness and standard kurtosis revealed that the data were normally distributed (standard skewness/standard kurtosis ≤±1.96).

### Group Differences in Demographic Variables

A one-way ANOVA was conducted to compare the demographic variables, including age, intellectual ability (IQ), and education level, in the four groups. There were significant differences in age [*F*(3,198)=3.87, *p*<0.05] and IQ [*F*(3,198)=12.99, *p*<0.001]. In terms of age, post-hoc comparisons using the Tukey HSD test indicated that the mean score for the TD group was significantly different from that of the comorbid group (*p*<0.05). As for IQ, post-hoc comparisons using the Tukey HSD test indicated that the mean score for the TD group (*M*=107.16; *SD*=15.91) was significant different from the RD (*p*<0.001; *M*=93.46; *SD*=13.72) and comorbid (*p*<0.001; *M*=93.06; *SD*=13.01) groups. The difference between the ADHD-I (*M*=102.21; *SD*=13.83) and RD (*M*=93.46; *SD*=13.72) groups was also significant (*p*<0.05). No significant difference was found in education level [*F*(3, 198)=0.66, *p*=0.58] (see Table 1).

**Table 1:** Demographic characteristics in ADHD-I, RD, ADHD-I+RD, and TD groups.

Variable	ADHD-I		RD		3. ADHD-I+RD		TD		*Post-hoc*
	(*n*=29)		(*n*=78)		(*n*=31)		(*n*=64)		
	*M*	*SD*	*M*	*SD*	*M*	*SD*	*M*	*SD*	
Age	9.57	1.15	9.15	1.00	9.68	1.34	9.01	0.97	4<3
IQ	102.21	13.83	93.46	13.72	93.06	13.01	107.16	15.91	4>2, 3
									1>2
Education level	3.34	0.81	3.12	0.81	3.17	1.00	3.16	0.89	–

A chi-square test was used to examine sex differences in each group. Our current study aims to examine the biological and physiological characteristics of our participants as males and females. There was a significant difference in sex [*χ*^2^ (3, 202)=8.67, *p*<0.05]. In the ADHD-I group, there were 69% males and 31% females. In the RD group, there were 53% males and 47% females. In the comorbid group, there were 81% males and 19% females. In the TD group, there were 56% males and 44% females (see Table 2).

**Table 2:** Results of chi-square analysis to examine sex differences across the groups.

	Total		ADHD-I		RD		ADHD-I+RD		TD		
	*N*=202		*n*=29		*n*=78		*n*=31		*n*=64		
	*n*	%	*n*	%	*n*	%	*n*	%	*n*	%	*χ* ^2^
Sex											8.67^*^
Male	122	60.4	20	69.0	41	52.6	25	80.6	36	56.3	
Female	80	39.6	9	31.0	37	47.4	6	19.4	28	43.8	

* *p*<0.05.

### MANCOVA Results in Three Domains of WM

The scores on the three types of WM were entered into a MANCOVA. After controlling for age and IQ, the multivariate main effects of ADHD-I [*F*(3, 170)=18.86, Pillai’s Trace=0.25, *p*<0.001, $\eta_{p}^{2}$=0.25], RD [*F*(3, 170)=3.26, Pillai’s Trace=0.05, *p*<0.05, $\eta_{p}^{2}$=0.05], and the interaction of ADHD-I×RD [*F*(3, 170)=9.26, Pillai’s Trace=0.14, *p*<0.001, $\eta_{p}^{2}$=0.14] proved to be significant. In the univariate analysis, there were significant main effects of ADHD-I on behavioral WM [*F*(1, 172)=53.98, *p*<0.001] and RD on verbal WM [*F*(1, 172)=8.45, *p*<0.05]. The main effect of RD on visual-spatial WM was marginally significant [*F*(1, 172)=3.32, *p*=0.07]. Furthermore, there were significant interaction effects between ADHD-I and RD in all three domains of WM: behavioral WM [*F*(1, 172)=13.04, *p*<0.001], verbal WM [*F*(1, 172)=5.97, *p*<0.05], and visual-spatial WM [*F*(1, 172)=5.27, *p*<0.05; see Table 3].

**Table 3:** Means, standard deviations, and results of 2×2 MANCOVA for three types of working memory, with age and IQ as covariates.

Variable	1. ADHD-I		2. RD		3. ADHD-I+RD		4. TD		Main effect				Interaction		Simple effects
	(*n*=29)		(*n*=78)		(*n*=31)		(*n*=64)		ADHD-I		RD		ADHD-I×RD		
	*M*	*SD*	*M*	*SD*	*M*	*SD*	*M*	*SD*	*F*	$\eta_{p}^{2}$	*F*	$\eta_{p}^{2}$	*F*	$\eta_{p}^{2}$	
Behavioral WM	22.14	2.43	19.83	3.58	25.18	3.67	18.29	4.34	53.98^***^	0.24	1.76	0.01	13.04^***^	0.07	4<2<1<3
Verbal WM	8.73	2.53	8.31	1.87	8.49	1.49	10.04	3.17	0.39	0.00	8.45^*^	0.05	5.97^*^	0.03	4<1, 2, 3
Visual-spatial WM^1^	19.98	7.14	22.22	5.95	20.63	8.21	17.31	6.01	2.46	0.01	3.32^†^	0.02	5.27^*^	0.02	4<1^†^, 2, 3^†^

* *p*<0.05;

*** *p*<0.001.

† =marginally significant.

1 =higher number of between errors, indicating a weaker ability in visual-spatial WM.

**Figure 1:**
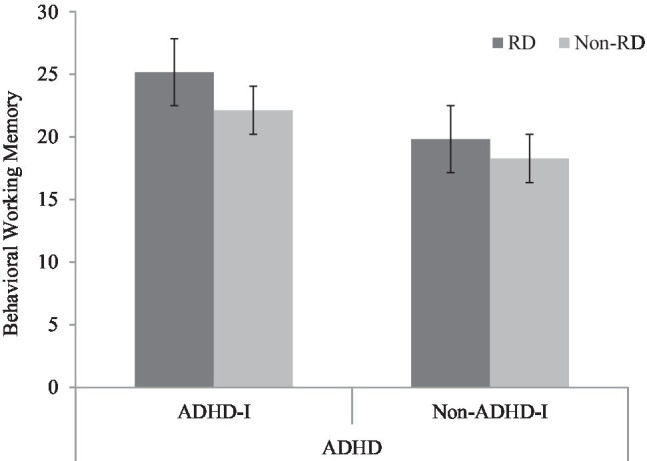
Interaction effect of ADHD-I and RD in behavioral working memory.

Given the significant interaction effects, simple effect analyses were conducted. The results revealed that the performance of the three disorder groups (ADHD-I: *p*<0.001; RD: *p*<0.05; comorbid: *p*<0.001) was significantly worse than that of the TD group in behavioral WM. Significant differences were also found between the RD and comorbid groups (*p*<0.001), between the ADHD-I and RD groups (*p*<0.001), and between the ADHD-I and comorbid groups (*p*<0.05) in behavioral WM. The bar chart shows that individuals with ADHD-I status, having RD, had an impact on behavioral WM, while individuals without ADHD-I, having RD, showed no difference in behavioral WM (see Figure 1).

**Figure 2:**
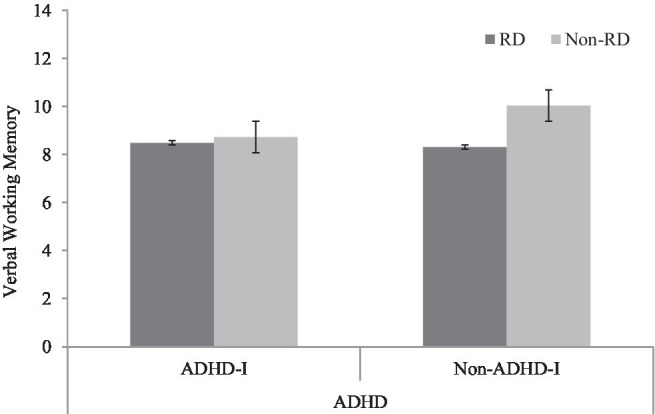
Interaction effect of ADHD-I and RD in verbal working memory.

In terms of verbal WM, the TD group significantly outperformed the ADHD-I (*p*<0.01), RD (*p*<0.01), and ADHD-I+RD (*p*<0.01) groups. No significant differences were found between the other three disorder groups (ADHD-I vs. RD: *p*=0.70; RD vs. comorbid: *p*=0.83; ADHD-I vs. comorbid: *p*=0.83). The bar chart illustrates that individuals without ADHD-I status had an impact on their verbal WM performance. In contrast, for individuals with ADHD-I, RD made no difference (see Figure 2).

**Figure 3:**
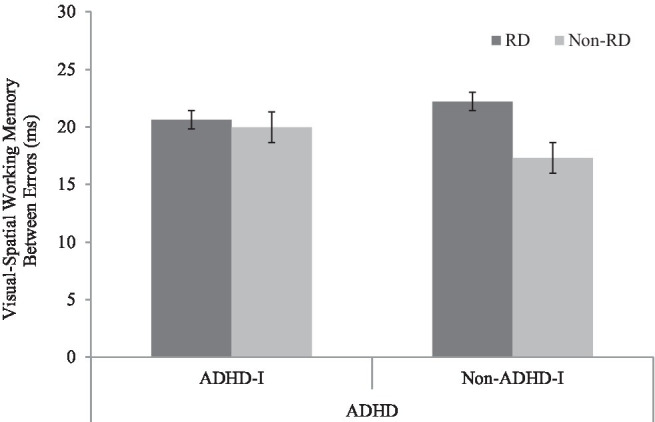
Interaction effect of ADHD-I and RD in visual-spatial working memory.

As for visual-spatial WM, the performance of the TD group was significantly better than that of the RD group (*p*<0.01). The differences between the TD and ADHD-I groups (*p*=0.076) and the TD and comorbid groups (*p*=0.086) were marginally significant. No significant differences were found between the other three disorder groups (ADHD-I vs. RD: *p*=0.13; RD vs. comorbid: *p*=0.39; ADHD-I vs. comorbid: *p*=0.79). The bar chart reveals that individuals without ADHD-I status, having RD, had an impact on their visual-spatial WM performance. Conversely, for individuals with ADHD-I, RD made no difference (see Figure 3).

## Discussion

Children with ADHD-I and RD typically show compromised WM abilities, which impacts their learning. Given the emergence of a wider range of validated measures for each of the main components of WM, there is now a need to provide a better understanding of the WM profiles associated with RD and ADHD-I and their comorbidity. This should employ both cognitive and behavioral measures. The current study compared the cognitive and behavioral measures of WM among the four groups and examined the impact of comorbidity on WM. Consistent with our hypotheses, we found that children with ADHD-I were associated with deficits in behavioral WM, while children with RD were associated with deficits in verbal WM and marginally associated with deficits in visual-spatial WM. As for the comorbid group, our findings showed an association with all three WM domains, especially severe impairment in behavioral WM, which supports the cognitive subtype hypothesis. Overall, the results indicate that ADHD-I and RD manifest distinct patterns of WM, which is in line with Kofler et al.’s (2019) conclusion that children with ADHD-I and RD could be attributed to a large proportion of the deficits in WM.

### Research Findings in the RD Group

Consistent with prior literature, children with RD were associated with a deficit in verbal WM. These findings corroborate Martinussen and Tannock’s (2006) observation that children with reading disorders exhibited impairments in the verbal domain of WM regardless of the presence of comorbidity with ADHD-I. Further, the current study used a backward digit span subtest, which requires participants to repeat the orally presented digits; this supported previous research showing that a WM deficit in RD is specific to the phonological loop (Ho et al., 2004; Kibby and Cohen, 2008; Chen et al., 2018). Phonological WM deficits, which are significantly associated with RD, have been reported in numerous studies (Szenkovits and Ramus, 2005; Hulme and Snowling, 2013; Lu et al., 2016). The present study showed that there was a marginally significant main effect of RD on visual-spatial WM. In fact, some studies found that children with RD exhibited poorer visual-spatial WM compared to other disorder groups, while other studies found that the visual-spatial WM performance of children with RD was relatively intact compared to children in the TD group (Pham and Hasson, 2014). One possible explanation for these inconsistent findings relates to the complex measures of visual-spatial WM. Our study used a computerized measure that assessed the participants’ ability to temporarily store and process information to successfully search for a hidden object; later studies may use more dynamic and comprehensive aspects of visual-spatial tasks, such as tracking visual sequences and transforming visual-spatial images, to examine the link between RD and visual-spatial WM (e.g., Logie, 1995; Duff and Logie, 1999; Alloway et al., 2006). Our findings also showed that RD was not associated with behavioral WM. One of the reasons for this non-significant finding is that the BRIEF rating scale was employed to measure general WM behavior within the home environment, instead of targeting reading-related behavioral WM. Researchers may wish to include other behavioral rating scales that have a stronger focus on reading-related WM measures in future research.

### Research Findings in the ADHD-I Group

Our findings in the ADHD-I group exhibited a main effect in behavioral WM but not in any of the cognitive WM assessments (i.e., verbal and visual-spatial). The ADHD-I group still exhibited comparatively poor cognitive and behavioral WM compared to the control group in our study. Such findings align with earlier studies that correlated ADHD-I with behavioral WM deficits (Wu et al., 2006; Brocki et al., 2007; Barkley and Murphy, 2010; Fuermaier et al., 2013a,b, 2015). Gioia et al. (2002) also revealed that children with ADHD manifested higher scores on the BRIEF scale compared to children with RD. As such, they argued that poor WM is a result of poor attentional control and inhibition. Poor behavioral inhibition is suggested to directly limit an individual’s WM, in addition to other important executive functioning abilities, such as self-regulation of affect-motivation arousal and internalization of speech (Barkley, 1997; Gray and Climie, 2016). Limited attentional control allows perceptual interference to directly impact the active maintenance of WM concerning a relevant piece of information (Lavie, 2005).

In the current study, ADHD-I was not associated with any significant weaknesses in the two cognitive WM measures. These findings are consistent with prior research that found slightly reduced or intact visual-spatial WM and verbal WM in the ADHD sample (Martinussen et al., 2005; Rhodes et al., 2012). This is perhaps because the phonological loop is intact in ADHD-I when tasks are more forgiving, such as when lists/stories are longer, allowing for brief fluctuations in attention. Another possible explanation is that stimulant medication may ameliorate visual-spatial and verbal WM in children with ADHD (Bedard et al., 2004; Shiels et al., 2008). Although information relating to medication usage was not provided by the participants in this study, previous research found that individuals with ADHD have a high prevalence of medication use (Russell et al., 2019). Future research should examine the role of stimulant medication in WM performance among Chinese children with ADHD-I.

### Research Findings in the Comorbid Group

With respect to the comorbid group, our study yielded interesting results. On the one hand, children with comorbidity of ADHD-I and RD had similar scores in verbal and visual-spatial WM compared to the pure groups. On the other hand, the score for behavioral WM was significantly higher in the comorbid group than in the other pure groups. It may be possible to conclude that children with pure RD or ADHD-I manifested distinct cognitive deficits, while both forms of deficit occurred together in the comorbid condition and produced an augmented clinical manifestation of behavioral WM deficits. These findings are consistent with a previous study conducted by Willcutt et al. (2005), who found that the comorbid group had worse performance in WM across the behavioral scales. One possible explanation could be the shared neural correlates between ADHD-I and RD. Langer et al. (2019) concluded that a combination of shared and distinctive brain alterations supported the multiple deficit model for ADHD-I and RD that the comorbid group showed greater impairments on all the same measures.

### Limitations and Conclusion

Some limitations of the present study should be noted. First, the dosage of ADHD medication taken by children with ADHD-I was not reported by the parents. Previous research reported that low doses of ADHD medication improve WM, whereas high doses impair WM in a group of children (Vance, 2008). Therefore, it would be interesting to compare WM performance between participants on high and low doses of ADHD medication in future research into Chinese children with ADHD-I. Second, given that there are other subtypes of ADHD (i.e., hyperactive/impulsive and combined types) and that the severity of symptoms varies across these subtypes, future research should explore whether ADHD symptoms would manifest differently in each of the WM domains. Third, given the relatively small sample size and unequal sizes across groups, the results should be confirmed with a larger Chinese sample of children with ADHD-I and/or RD. Fourth, this is the first study to compare different WM profiles using cognitive assessment batteries and the behavioral rating scale. In the present study, the comorbid group of children with ADHD-I+RD exhibited considerable deficits in behavioral WM, which was consistent with the view that ADHD is heterogeneous in nature (Luo et al., 2019). Behavioral assessment does not require any training prior to the use of psychometric scales and is valuable in identifying children at risk of poor WM (Alloway et al., 2010). The BRIEF rating inventory (Gioia et al., 2000) illustrates both home and school environments in which WM failures occur. It provides an initial step in identifying possible WM deficits prior to performing any additional cognitive tests (Alloway et al., 2010). Although comprehensive cognitive and behavioral assessments could assist in identifying specific structural or functional areas of WM on which to focus intervention, future research should also include WMRS. Understanding the scope of each type of assessment is crucial for the timing of the administration of cognitive functioning tests (Hartman and Blough, 2013). Finally, the current study reported a significant difference in the sex ratio among the four subgroups. In particular, more males were identified in the ADHD-I (69%) and comorbid (81%) groups. Sex differences in the prevalence of ADHD-I and comorbid diagnoses are well documented in the literature (Ramtekkar et al., 2010; Willcutt, 2012; Skogli et al., 2013), yet the underlying reasons for the observed differences remain to be investigated (Elkins et al., 2011; Kan et al., 2013). Although in-depth exploration is beyond the scope of this paper, there is evidence supporting sex disparity in WM, especially when WM is deconstructed into spatial and verbal components. Further research is therefore warranted to understand whether there are sex-based differences in the WM domains in children with ADHD-I and ADHD-I+RD groups (Pham and Hasson, 2014).

Despite these limitations, our findings have potential implications. The present study was a response to earlier calls for targeted interventions for ADHD-I, RD, and comorbid groups of children. These include computerized training targeting WM, which has been associated with a reduction in ADHD symptoms and an improvement in the visual domains (e.g., Flak et al., 2019). For example, Klingberg et al. (2005) tested the effectiveness of a five-week WM training program on students with ADHD and found that it significantly improved their WM capacity and behaviors. In addition, Luo et al. (2013) conducted computerized WM training focused on visual-spatial WM, verbal WM, and central executive tasks for children with RD. They found that intensive and adaptive computerized WM training contributed to a gradual increase in WM capacity. In particular, they observed a significant improvement in visual rhyming and reading fluency tasks. Another alternative would be to supplement verbal instruction with visual aids and demonstrations for children with RD.

In conclusion, this study indicated that children with ADHD-I and/or RD exhibited diverse cognitive profiles, particularly in the Chinese population in Hong Kong. Children with comorbid symptoms demonstrated poorer performance in behavioral WM than those in the pure groups. Furthermore, children in the RD group demonstrated weaker ability in verbal WM and visual-spatial WM than did the pure groups. These findings provide a clear picture of the distinctive WM profiles across different groups, allowing for the development of intervention programs in the future.

## Data Availability Statement

The original contributions presented in the study are included in the article/supplementary material, and further inquiries can be directed to the corresponding author.

## Ethics Statement

The studies involving human participants were reviewed and approved by the Human Research Ethics Committee, The Education University of Hong Kong. Written informed consent to participate in this study was provided by the participants’ parents.

## Author Contributions

KP was responsible for the conception of the research questions, study design, data interpretation, and writing. MH was responsible for data analysis and writing. L-CW was responsible for providing input on this paper. All authors have agreed the final version of the manuscript.

## Funding

This work was partially funded by the Block Grant Faculty Fund from the Faculty of Education and Human Development at The Education University of Hong Kong. The funders had no role in study design, data collection and analysis, decision to publish, or preparation of the manuscript.

## Conflict of Interest

The authors declare that the research was conducted in the absence of any commercial or financial relationships that could be construed as a potential conflict of interest.

## Publisher’s Note

All claims expressed in this article are solely those of the authors and do not necessarily represent those of their affiliated organizations, or those of the publisher, the editors and the reviewers. Any product that may be evaluated in this article, or claim that may be made by its manufacturer, is not guaranteed or endorsed by the publisher.
